# Interplay Between Microbiota, Toll-Like Receptors and Cytokines for the Maintenance of Epithelial Barrier Integrity

**DOI:** 10.3389/fmed.2021.644333

**Published:** 2021-05-28

**Authors:** Iaroslav Semin, Justus Ninnemann, Marina Bondareva, Ilia Gimaev, Andrey A. Kruglov

**Affiliations:** ^1^German Rheumatism Research Center (DRFZ), a Leibniz Institute, Berlin, Germany; ^2^Belozersky Institute of Physico-Chemical Biology and Biological Faculty, M.V. Lomonosov Moscow State University, Moscow, Russia; ^3^Center for Precision Genome Editing and Genetic Technologies for Biomedicine, Engelhardt Institute of Molecular Biology, Russian Academy of Sciences, Moscow, Russia

**Keywords:** TNF, TLR4, cytokine, intestinal barrier, inflammation

## Abstract

The intestinal tract is densely populated by microbiota consisting of various commensal microorganisms that are instrumental for the healthy state of the living organism. Such commensals generate various molecules that can be recognized by the Toll-like receptors of the immune system leading to the inflammation marked by strong upregulation of various proinflammatory cytokines, such as TNF, IL-6, and IL-1β. To prevent excessive inflammation, a single layer of constantly renewing, highly proliferating epithelial cells (IEC) provides proper segregation of such microorganisms from the body cavities. There are various triggers which facilitate the disturbance of the epithelial barrier which often leads to inflammation. However, the nature and duration of the stress may determine the state of the epithelial cells and their responses to cytokines. Here we discuss the role of the microbiota-TLR-cytokine axis in the maintenance of the epithelial tissue integrity. In particular, we highlight discrepancies in the function of TLR and cytokines in IEC barrier during acute or chronic inflammation and we suggest that intervention strategies should be applied based on the type of inflammation.

## Introduction

The intestinal barrier represents a complex system of epithelial cells, Paneth cells, goblet cells, infiltrating immune cells, mucus, immunoglobulin A (IgA) antibodies and antimicrobial peptides (1). Underneath the epithelial cells multiple immune cell subsets are localized, which contribute to the maintenance of the border between the host and the microbiota. Disturbance of this barrier by extrinsic and intrinsic factors may result in the influx of various bacterial products inside of the host body leading to chronic inflammatory reactions. Such stimuli include dietary components, commensal microflora or invading pathobionts from the environmental side. Moreover, genetic variability of the host and adaptive immune response toward these stimuli may also influence barrier integrity.

The main component of the intestinal barrier is a layer of epithelial cells that forms the very first physical border between the host organism and its external surroundings, which could be potentially detrimental for the host cells. These epithelial cells are tightly connected with each other to ensure proper control of the molecules that enter the body from the intestinal fluids. The junctional complex of intestinal epithelial cells is composed of the three main different types of connections—tight junction (TJ), adherence junction and desmosome. Tight junctions between epithelial cells are facilitated by a set of proteins [Claudin, ZO1, Occludin, F-actin, Myosin, Myosin light chain kinase (MLCK)], which form together an apical junctional complex in order to seal the paracellular space between epithelial cells. There are two additional zones of cell-to-cell contacts beneath TJ named “Adherence junction” and “Desmosome.” They consist of E-cadherin, α-catenin 1, β-catenin, catenin- δ1 and desmoglein, desmocollin, desmoplakin, respectively (2). Together they provide cell-to-cell and cell-to-matrix connections and create a paracellular space. Normal gut permeability facilitates paracellular transport of nutrients, water and essential solutes. Disruption of such TJ may result in the penetration of various molecules and microorganisms, leading to inflammation.

The whole spectrum of cell types within the gut epithelium develops from the epithelial stem cells located at the base of the crypts. Stem cells give rise to distinct cell types of the intestinal epithelium: absorptive cells (enterocytes) and secretory cells (goblet, Paneth, enteroendocrine, and tuft cells). Fate decision toward the absorptive phenotype is critically dependent on the NOTCH pathway (3). Genetic and pharmacological manipulation of NOTCH signaling also revealed its crucial role in the maintenance of the epithelial stem cell niche (4–6). Apart from NOTCH, wingless and Int-1 (WNT) signaling plays an essential role in epithelial stem cell functions influencing functioning of different transcription factors including Ascl2, sox9, Lgr5 (7–9).

## Regulation of The Epithelial Cell Functions During Homeostasis

**Figure 1:**
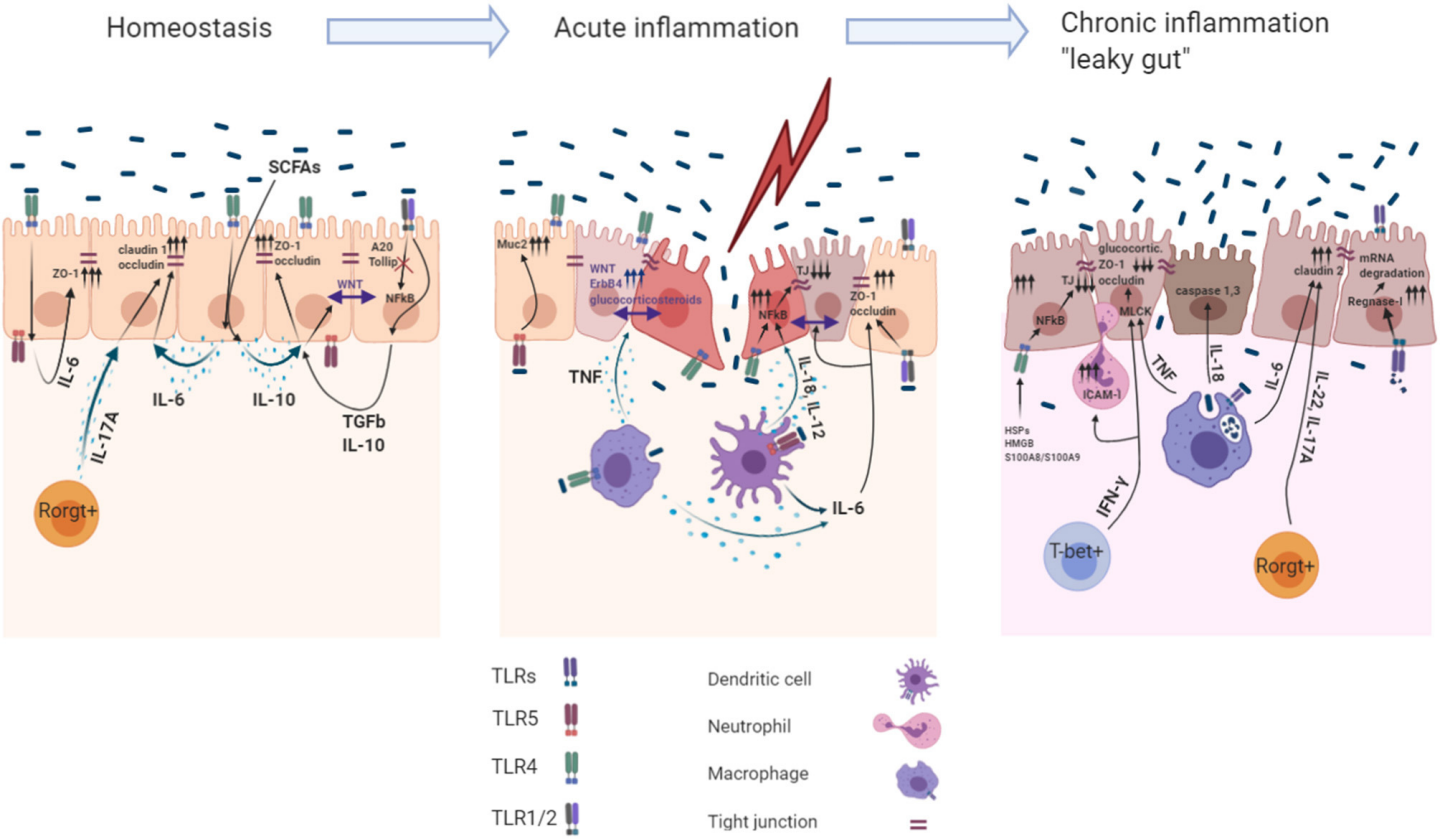
Role of the TLR induced cytokines in acute and chronic intestinal inflammation. The intestinal mucosa is separated from the body immune environment by a single layer of the intestinal epithelial cells (IECs) that provides a physical and functional barrier. Beneath the IECs immune cells reside in the *lamina propria*, maintaining the intestinal tissue at the hyporesponsive state. Intestinal immune homeostasis: Constant recognition of microbiota by TLR4 and TLR1/2 leads to IL- 6, IL−10, TGF-β1 production within IECs. Autocrine recognition of these cytokines maintains IEC barrier integrity by promoting expression of the TJ proteins (ZO-1, claudin-1, occludin). Moreover, action of the IL-10 induce Wnt signaling within IECs, which maintains their proliferation. A20 and Tollip are the main inhibitors of the TLR1/2 signaling facilitating the avoidance of undesired response toward microbiota. Rorgt+ cells during homeostasis produce IL-17A to maintain constant production of claudin-1 and occluding within IECs. Acute inflammation: Sensing microbiota within the *lamina propria* induces production of pro-inflammatory cytokines and cytoprotective factors via NFkB dependent mechanism. Basolateral TLR5 mediated recognition of bacteria leads to MUC2 production in IECs. IL-6, TNF production by M?s and DCs during acute inflammation enables barrier repair program within IECs. TNF induced production of the glucocorticoids and ErbB4 receptor tyrosine kinase in IECs induce tissue repair functions and resolve late stages of the acute inflammation. Pro-inflammatory cytokines IL-18, IL-12 involved in IEC barrier dysfunction by downregulating TJ proteins (ZO-1, occludin). TLR1/2 basolateral recognition of bacteria promotes ZO-1/occluding expression in IECs. Chronic inflammation: Chronic TNF sensing by IECs reduces their ability to migrate toward crypts villi, modulates MLCK which decreases claudin-1, ZO-1, and ZO-2 expression and decreases glucocorticoids synthesis, which is indispensable for later inflammation resolution. IFN-γ activates the expression of ICAM-1 which resulted in increased IEC barrier permeability caused by neutrophil migration into subepithelial layers and paracellular space via modulation of MLCK. TLR4 dependent recognition of the HSPs, HMGB1 and S100A8/S100A9 by IECs leads to downregulation of the expression of ZO-1.

In steady state, a delicate balance is maintained between bacterial composition, the mucosal immune system and the intact epithelial barrier. Commensal microbiota is transported in a highly controlled manner to be recognized by the immune system in the gut-associated lymphoid tissues (2). Due to the non-pathogenic nature of such microorganisms, the immune system responds with the production of non-inflammatory cytokines, such as TGF-β1, IL-10 and cytokines which are important for the IEC barrier, like IL-22 (Figure 1). Both mutation of IL-10 pathway in humans and the genetic ablation of *Il10* resulted in development of intestinal inflammation demonstrating a crucial role for IL-10 in the tolerance maintenance and barrier integrity (10). Although *Il10*^−/−^ mice are not defective in mucin production, but have its defective loose quality that makes mice suffer from spontaneous colitis (11). Similarly, TGF-β1 directly modulates TJ protein expression (12, 13), significantly decreasing JNK-pathway activation and protects cells from TNF-mediated downregulation of occludin and ZO-1 (14). IL-22 controls not only the expression of TJ proteins (15), but also the expression of various antimicrobial proteins. IL-22 deficient animals exhibited defects in IEC barrier (15) and failed to repair IEC functions in multiple inflammatory models linked to the disruption of the IEC barrier. IL-22 was further reported as a necessary cytokine for TJ formation and mucin production (16). Patients with HIV infections have decreased IL-22 levels and concomitantly impaired IEC barrier and increased bacterial translocation (16). Interestingly, the natural antagonist of IL-22 (IL-22BP; IL-22Ra2) which regulates the biological actions of IL-22 was found to be expressed by various immune cells (17). Recent data suggested that type III innate lymphoid cells (ILC3) instruct a special subset of dendritic cells in the isolated lymphoid follicles to produce IL-22BP via lymphotoxin (LTα_1_β_2_)–lymphotoxin β receptor (LTβR) interaction (18), revealing a novel mechanism of the epithelial barrier control in steady state and during inflammation.

Commensal microbiota produces multiple “non-self” ligands and IECs recognize such molecules and tune their transcriptional program to keep the barrier tight. There are several families of receptors sensing various microbial products: Toll-like receptors (TLR), NOD-like receptors (NLR), RIG-like receptors (RLR), and others (19). TLRs are widely expressed on the epithelial cells in the small and large intestine and their expression is tightly regulated in order to ensure the proper innate immune recognition. Mostly, TLRs are expressed among the whole IEC lineage: absorptive enterocytes (20, 21), stem cells (22), enteroendocrine cells (23), goblet cells (24, 25), Paneth cells (26–28), and micro-fold cells (29, 30). The distribution pattern of TLR expression on epithelial cells varies among the intestinal tract. Price et al. recently provided an elegant analysis of TLRs expression in the large and small intestine of mice (27). It was shown that TLR2, TLR5, and TLR9 are more restricted to the small intestine when TLR2, TLR4, and TLR5 are upregulated in the colonic epithelial cells. In addition, TLR signaling is controlled by the polarized expression on the cell surface. For instance, TLR2 and TLR4 are expressed at low levels on basolateral sides of IEC in the small intestine, while TLR5 is expressed mainly on basolateral sides of the colon (31). Furthermore, apical TLR9 recognition of CpG oligonucleotides prevents NFkB translocation into the nucleus and limits inflammatory response.

The tuning of the immune responses via IEC-derived TLRs is achieved by several mechanisms. Epithelial cells modulate TLR receptor-ligand interactions by the downregulation of the receptor expression (32) or by translocating receptors from apical to basolateral sides or to lysosomes (33–36) to avoid excessive sensing of bacterial products. Indeed, overexpression of *Tlr4* on epithelial cells resulted in the overactivation of TLR4 pathway in IECs that lead to the increased production of IgA by plasma B cells (37). This loop potentially demonstrates a regulatory mechanism where IgA antibodies after being induced neutralize excessive bacteria-TLR4 interaction (20). Next, expression of molecules downstream of TLRs is modulated in IEC via various posttranslational modifications like glycosylation, phosphorylation, and ubiquitination (38, 39). Finally, IECs were reported to bind and modify immunogenic parts of MAMPs in order to diminish ligands property to induce signals (40, 41).

Apart from this, TLRs are involved in crypt dynamics control. For instance the depletion of MyD88 or TLR2 was associated with an abrogation of trefoil factor 3 (TFF3) expression, which is required for goblet cells maturation (24). Furthermore, TLR4 was shown to mediate NOTCH expression implying that TLRs may interfere with processes of stemness and differentiation in the stem cell niche (1). However, the role of TLR4 on stem cell differentiation remains controversial (42, 43). Deletion of TLR1 or TLR5 in mice induced the loss of the mucus layer integrity via impaired MUC2 production in goblet cells (1). Moreover, ablation of the TLR recognition by MyD88 deletion abrogated the production of antimicrobial peptides RegIIIβ and RegIIIγ by goblet cells in mice (20). Thus, sensing of bacterial products via TLRs modulates mucus layer permeability that limits the direct interaction of commensals with the epithelium and induction of spontaneous inflammation (33, 44).

Altogether, IEC barrier exerts multiple strategies to avoid activation of inflammatory pathways in normal conditions via cytokine production and regulation of TLR signaling to maintain its integrity.

## Regulation of The Epithelial Cell Functions During Acute Inflammation

Disruption of the cell-to-cell contacts at the epithelial layer leads to increased bacterial products penetration, which triggers inflammatory immune responses. The nature of the damage may further define the type of immune response and subsequent immune reactions driving IEC repair (Figure 1). Epithelial barrier disruption may be induced by acute stimuli, such as ingestion of toxic substances (oxazolone, dextran sodium sulfate etc.) (45), by physical force or by the invasion of various pathogens, such as *Clostridium difficles, Citrobacter rodentium, Salmonella enterica* etc. These acute stimuli result in IEC layer erosion, the influx of commensal bacteria and activation of the innate arm of the immune system (46), while the chronic reduction of the barrier leads to the mobilization of both arms of the immune system as well as the genomic instability of epithelial cells (47). In case of the acute damage of the epithelium caused by pathogens, the immune system should eliminate the causing agent or pathogen, while ensuring the proper restoration of the barrier. Thus, the gut immune system is determined to restore the barrier functions in both acute and chronic settings, but triggers are different and, thereby, advocate for different intervention strategies.

Eliciting a protective immune response is required for the successful restoration of the barrier during bacteria-induced colitis. Here TLR-proinflammatory cytokine module is instrumental for the clearance of the inflammatory triggers and it is also involved in further tissue repair processes. Indeed, there are multiple examples of protective functions of TLR receptors in this setting. For instance, TLR1 is found to be crucial for the protection during acute intestinal inflammation induced by *Yersinia enterocolitica* in mice and the maintenance of the increased IEC barrier permeability (48). TLR5 was reported to limit intestinal colonization with vancomycin-resistant Enterococcus (VRE) by the induction of RegIIIγ expression (49) and IEC-derived TLR5 mediates production of IL-6 and IL-12 by CD11c^+^ in response to Salmonella enterica infection (50). The significance of TLR/MyD88 signaling pathway for the recovery of IECs was also shown during acute colitis induced by *Helicobacter hepaticus* or *Citrobacter rodentium* (51). Furthermore, *Myd88*^−/−^, *Tlr1*^−/−^, *Tlr2*^−/−^ mice were characterized by the early loss of tight junctions and diminished transepithelial resistance during acute intestinal inflammation (52).

Apart from IEC barrier disruption by pathogens, there is a significant amount of the research directed toward the dissection of the pathways which are crucial for IEC barrier restoration during injury caused by chemical agents, such as DSS, oxazolone and others. Herein the inflammation is caused by the influx of commensal microbiota in the intestinal tissue. Thus, TLR signaling pathways and pro-inflammatory cytokines facilitate the inflammation that is needed for the clearance of the bacteria and may possess protective functions. Consistently, seminal work from Medzhitov's lab showed the crucial role of TLR4/MyD88 signaling for the maintenance of the intestinal homeostasis and barrier repair during acute DSS colitis in microbiota dependent manner (53). Activation of TLR4 signaling pathways was crucial for the clearance of commensal bacteria by infiltrating innate immune cells (54). In contrast, several other studies highlighted the pathogenic function of TLR4 signaling in DSS colitis. In particular, an increase of *E. Coli* in the microbiota was associated with less severe colitis in TLR4 deficient mice (55). LPS, main TLR4 agonist, also may induce epithelial damage *in vitro* and *in vivo* via excessive phosphorylation of the focal actin kinase (FAK) in TLR4/MyD88 dependent pathway in epithelial cells (56). Using an ileal cell line, LPS was further reported to be instrumental in the induction of paracellular permeability via ZO-1 and occludin downregulation via TLR4 (57). Interestingly, LPS serotypes differentially affect inflammatory cytokines expression *in vitro*. Among others, LPS from *S. marcescens* has the most pronounced effect on the reduction of transepithelial electrical resistance. That correlated with an increase in NFkB activation, IL-8 production as well as TNF (58). Furthermore, *E. coli* LPS, but not LPS from *B. dorei*, influenced the incidence of autoimmune diabetes in non-obese diabetic mice and correlated with the development of autoimmunity in humans (59). Therefore, the role of TLR4 during acute IEC disruption is determined by the microbiota composition and therapeutic strategies targeting TLR4 should be considered given the prevalence of various microorganisms and pathogens in individual contexts.

TLR signaling mediates the production of multiple pro-inflammatory cytokines, among them TNF, IL-6, and IL-1β. TNF a cytokine with pleiotropic functions in the body is of particular significance in this context. On the one hand, TNF is crucial for the host defense against intracellular pathogens (60) but on the other hand it drives multiple autoimmune pathologies associated with a reduction of the epithelial barrier, such as inflammatory bowel disease (IBD), ankylosing spondylitis and rheumatoid arthritis. Importantly, anti-TNF therapy is highly effective in the treatment aforementioned autoimmune pathologies (61). Despite the tremendous success of the TNF blockade, a significant proportion of patients do not respond to this type of biological interventions further highlighting the heterogeneity of given autoimmune conditions and pleiotropy of TNF itself. It is worth mentioning that TNF exerts its functions via two receptors, TNFR1 and TNFR2 (62) inducing distinct transcriptional programs. TNF plays a protective role during acute colitis induced by DSS, as TNF deficient mice and anti-TNF therapy in wild type mice during colitis resulted in severe inflammation (63). Short acute IEC exposure to TNF induced glucocorticoid synthesis and, thereby, ameliorated the late stages of DSS colitis (64). Furthermore, TNFR1 mediated protective functions, while TNFR2 was deleterious upon acute disruption of epithelium (65). Apart from the induction of anti-inflammatory mediators that are crucial for the barrier restoration, TNF also contributes to the restoration of the epithelial barrier via modulation of Wnt (66). TNF administration during acute DSS colitis promoted the intestinal cell survival and restitution via elevating expression the ErbB4 receptor tyrosine kinase (67). In addition, another study conducted on the IL-10 deficient mice colitis model suggested that the binding of TNF by TNFR1 and following Il1b upregulation is essential for the early defensive response within colonic epithelial cells (68, 69). Kuhn et al. showed that *Bacteroidales spp*. induced IL-6 secretion by IECs in a MyD88-dependent manner, while *Il6*^−/−^ mice were more susceptible to *Citrobacter rodentium* infection and had a thinner mucus layer, as well as decreased claudin-1 expression (70). Finally, IL-6 also activated NOTCH dependent program of IEC barrier restoration during acute DSS colitis (71).

Thus, proinflammatory cytokines exert its protective functions during acute barrier injury to facilitate efficient clearance of invading microorganisms.

## Regulation Of the Epithelial Cell Functions During Chronic Inflammation

Various extrinsic factors, such as the environment, particular diet, and exposure to hazardous chemicals, may result in the chronic elevation of pro-inflammatory cytokines and the reduction of the gut permeability for a long period of time (Figure 1). The state of an increased gut permeability and the perturbation of local immunity in the gut is called “leaky gut.” This phenomenon has been described not only in IBD patients, but also in many metabolic and autoimmune disorders. “Leaky gut” syndrome is characterized by an impaired mucin synthesis, a decreased expression of junctional proteins and epithelial cell death. Importantly, increased permeability of the epithelium is often found before the development of clinical symptoms (72).

Taking into account the fundamentally different nature of IEC barrier reduction during acute and chronic stress, it is plausible that TLR and cytokines may have distinct, and even opposing functions depending on the duration of inflammation. Consistently, deep analysis of the mutational landscape from inflamed IBD tissue and corresponding non-inflamed parts revealed mutations in several genes, such as *NFKBIZ, ZC3H12A (Regnase-1)* and *PIGR*. Interestingly, Regnase-1 is activated in response to TLR stimulation and degrades mRNA of many downstream immune signaling genes (47), including PIGR (73), NFKBIZ (74), and members of the IL-17 pathway (75). Furthermore, DNA methylation patterns and transcriptional program in IECs differed between healthy and IBD patients (76). Chronic exposure of IECs to TNF exclusively affected their migration from the crypt to the villus (77). In addition, chronic inflammation modeled by long-term culture of colonic organoids in the presence of TLR agonists and pro-inflammatory cytokines resulted in chronic NFkB activation and the transformation of epithelial cells. Finally, organoid cultures from IBD patients showed an inflammatory phenotype with decreased size and budding capacity and inverted polarization (78). Altogether, these data suggested that chronic inflammation might transform the genetic program and the functions of IECs and their ability to maintain the epithelial barrier.

Chronic subclinical inflammation is characterized by an increase in cytokine production and in release of endogenous TLR4 ligands. In particular, high mobility group box 1 (HMGB1) protein, the heat shock proteins and calcium binding protein A8 and A9 (S100A8/S100A9) (79) are released during an inflammation and chronic conditions, like metabolic disorders (80). Their binding to TLR4 leads to the secretion of the pro-inflammatory cytokines IL-1β, TNF, IL-6, IL-17A, IL-18, and IL-12 in the intestine (31, 81). Furthermore, TLR4 activation within the gut epithelium is associated with the activation of myosin light chain kinase (MLCK), which reduces the tight junction of IEC barrier and may lead to the development of “leaky gut” (82–84).

As mentioned earlier, increased gut permeability may be induced by extrinsic factors, like diet, environmental factors but also by intrinsic factors, such as elevated levels of pro-inflammatory cytokines (85, 86). In particular, TNF, IL-6 and IFN-γ are associated with the epithelial barrier impairment and increased gut permeability (31, 87–89). These cytokines once produced chronically may significantly reduce IEC barrier. So IFN-γ was found to modulate the expression of the neutrophil adhesion molecule ICAM-1, which resulted in increased permeability and the migration of neutrophils into the subepithelial layers and paracellular space (90). Apart from this, IFN-γ enhanced Th1 immune responses and also increased CD14 and TLR4 expression, as well as LPS uptake by IECs (86). For instance, IL-6 increased permeability-promoting tight junction protein (claudin-2) in colonic cell culture via activation of c-Jun N-terminal kinase (JNK) pathway (91). IEC stimulation with TNF lead to the upregulation of the MLCK, phosphorylation of myosin II light chain (MLC) and the subsequent decrease in barrier integrity. Furthermore, TNF induced the loss of ZO-1 and occludin expression and decreased trans-epithelial electrical resistance (92). In immune-mediated colitis model, it was further shown that TNFR2 pathway, but not TNFR1 signaling, increases MLCK expression resulting in tight junction dysregulation, barrier loss, and more severe disease (93). Chronic exposure to TNF, in contrast to acute stimuli, actually decreased glucocorticosteroid production and perpetuated inflammation (94). Given multiple effects of TNF on the intestinal biology, it is predicted that anti-TNF therapy restores the intestinal barrier in many autoimmune diseases (95). It has been shown in several reports that anti-TNF therapy directly influenced tight junction protein expression (96), while others showed the restoration of EC survival rate (97). *In vitro* experiments also indicated that sera from IBD patients directly regulates ZO-1 and occludin expression in IECs via TNF. Moreover, TNF was further shown to downregulate claudin-1, claudin-2, claudin-4, and occludin expression in IECs layer (95). Interestingly, IL-6 promoted crypt organoid proliferation stem cell numbers (98). Furthermore, anti-IL-6 therapy in IBD patients ameliorated the disease, but increased the risk of developing GI abscesses and perforation (99), suggesting that IL-6 contribute to inflammatory processes, but also may maintain epithelial barrier. Thus, upon chronic inflammatory stimuli epithelial cells modify their transcriptional program, expression patterns of receptors and, thereby, may respond differently toward pro-inflammatory cytokines.

## Conclusions

IEC barrier integrity is maintained not only by a complex system of tight junction proteins and strict compartment-dependent distribution of TLRs on apical and basolateral sides of IECs but also by a network of immune cells that mediate cell proliferation and epithelial permeability via cytokines. In a healthy state IECs exhibit multiple mechanisms that dampen TLR-dependent recognition of the microbiota. During acute injury of IEC barrier by chemical agents or pathogens the TLR-TNF axis is triggered toward the clearance of the pro-inflammatory stimuli and further drives IEC layer restoration via activation of the glucocorticosteroid synthesis, WNT pathway and ErbB4 kinase. In contrast to acute damage, chronic inflammation induces genetic instability, changes of methylome, transcriptome and the polarity of TLRs expression in IECs. This results in their modified response toward TLR agonists and TNF. Thus, the character and duration of inflammation should be considered for the modeling of studies aiming to dissect the mechanisms of IEC barrier integrity during various injury.

## Data Availability Statement

The raw data supporting the conclusions of this article will be made available by the authors, without undue reservation.

## Author Contributions

IS, MB, JN, IG, and AK analyzed the literature and studies and wrote the manuscript. All authors contributed to the article and approved the submitted version.

## Conflict of Interest

The authors declare that the research was conducted in the absence of any commercial or financial relationships that could be construed as a potential conflict of interest.
